# Management of high-grade pediatric renal trauma in tertiary referral hospital in Indonesia: A case series and literature review

**DOI:** 10.1016/j.ijscr.2024.109671

**Published:** 2024-04-21

**Authors:** Soetojo Wirjopranoto, Yufi Aulia Azmi, Kevin Muliawan Soetanto

**Affiliations:** a Department of Urology, Faculty of Medicine, Universitas Airlangga-Dr. Soetomo General Academic Hospital, Surabaya, Indonesia; b Department of Health Sciences, University of Groningen, University Medical Center Groningen, Groningen, the Netherlands; c Department of Immunology, Faculty of Medicine Siriraj Hospital, Mahidol University, Bangkok, Thailand

**Keywords:** Conservative, Nephrectomy, Mortality, Pediatric, Surgical intervention

## Abstract

**Introduction and importance:**

Genitourinary tract injuries constitute 10 % of all traumas, with renal injuries being common in pediatric cases due to reduced perirenal fat and abdominal wall muscle development. However, very few reports of pediatric renal trauma in Indonesia have been reported. In this case series, We present a case series of high-grade renal injury and review the literature on pediatric renal trauma in Indonesia.

**Case presentation:**

We present four cases with renal trauma as the subjects of this case study. The 13-year-old boy, who was the initial patient, complained of hematuria and abdominal pain after falling from a tree. The second patient, the 13-year-old boy, presented with left lower back pain and hematuria after being elbowed in the left waist. The third patient, a 14-year-old boy, had been in a motorcycle accident and got grade 5 renal injury according to AAST classification. The final case involved a 4-year-old boy who experienced recurrent hematuria caused by a pseudoaneurysm following blunt renal trauma.

**Discussion:**

Trauma is the leading cause of morbidity and mortality in children, with approximately 3 % of children assessed in pediatric hospital trauma departments having had trauma. With appropriate management according to guidelines, mortality can be avoided.

**Conclusion:**

The case series highlights the significance of treating pediatric renal trauma patients individually according to their hemodynamic state and degree of impairment.

## Highlights

• Non-operative management is safe and effective for hemodynamically stable children with blunt renal trauma.
• Surgical intervention such as nephrectomy is often required in hemodynamically unstable high-grade renal trauma in pediatric patients.
• Management according to the guidelines will provide optimal results.

## 1 Introduction

Injuries to the genitourinary tract make up 10 % of all traumas, with the kidney being the most frequently affected site, accounting for over half of all urinary tract injuries [1]. Renal injuries are the most common form of pediatric genitourinary trauma. Compared to the adult kidney, the pediatric kidney is believed to be more vulnerable to trauma for several reasons. Less perirenal fat and underdeveloped abdominal wall muscles protect the pediatric kidney. The pediatric kidney also sits lower, so the rib cage less protects it. In addition, the pediatric vertebral column may be more pliable, leading to more stretch injuries on the ureters. Approximately 5 % to 20 % of pediatric patients with blunt trauma will have renal trauma [2].

According to an analysis of the US National Trauma Data Bank, patients under the age of 19 accounted for almost 25 % of all kidney injuries [3]. Most blunt trauma-related kidney injuries fall into the low-grade category and respond well to conservative treatment [4,5]. 90 % were blunt trauma injuries, with almost 80 % grade 1 to 3 injuries. Around 20 % of patients with grade 4 injuries underwent an open operation, and 50 % of those with grade 5 injuries underwent open surgery [6]. Minimally invasive treatments such as angioembolization or ureteral stents were seen in about 5 % of all cases, with 10 % to 15 % for grade 4 to 5 injuries. Nephrectomy for pediatric renal trauma is rare, occurring in around 5 % of admissions [1].

Most pediatric renal trauma is managed nonoperatively, which is a strong recommendation by the EAU pediatric urology guidelines. The degree of renal injury in children can be determined by applying the American Association for the Surgery of Trauma (AAST) organ injury severity scale, which also allows for the classification of the mode of injury as either blunt or penetrating [7]. Patients with grades 4 and 5 renal injuries are more likely to receive an intervention. Intervention is required if children are hemodynamically unstable at any point in their presentation [8]. The interventions most often needed are endoscopic or percutaneous, such as percutaneous nephrostomy tube, ureteral stent, percutaneous drain placement, and angioembolization. Although case series had been previously reported from Malang and Surabaya, there still have been very few reports of pediatric renal trauma in Indonesia [9,10]. We present a case series of isolated high-grade (grade 4 and 5) renal injuries focused on the management in our center, Surabaya, and a literature review. The present study was reported to comply with the PROCESS criteria [11].

## 2 Case presentation

### 2.1 First patient

**Fig. 1:**
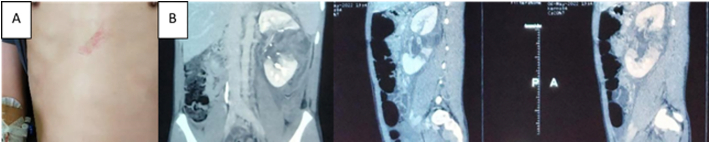
(A) Physical examination revealed a hematoma in the epigastric region; (B) Computed tomography (CT) scan revealed a total left renal rupture with avulsion of the renal hilum and devascularisation of the medial pole.

A 13-year-old boy presented with abdominal pain with a dull sensation focused in the flank region that progressively worsened and hematuria after falling approximately 3 m from a tree and landing in a slumped position. Physical examination revealed a hematoma in the epigastric region (Fig. 1A). Laboratory tests showed mild anemia with a hemoglobin (Hb) level of 8.4 g/dL, elevated leukocyte count of 19,250 × 103/uL, and a hematocrit of 31.6 %. The patient received transfusion therapy containing two packed red blood cell units, increasing Hb to 10.6 g/dL. Urinalysis revealed a urine pH 7.5, erythrocytes +3, and leukocytes +1. From radiological examinations, free fluid was discovered in Morrison's pouch during a focussed assessment using sonography for trauma (FAST) ultrasound examination. A computed tomography (CT) scan revealed a total left renal rupture with avulsion of the renal hilum and devascularisation of the medial pole (Fig. 1B) assessed with renal trauma AAST grade V. The patient was managed conservatively with bed rest for 14 days. The patient was discharged and scheduled for regular monthly follow-ups, with the option to visit the outpatient clinic if any complaints arise. The patient has not reported any issues as of the time of writing this paper.

### 2.2 Second patient

**Fig. 2:**
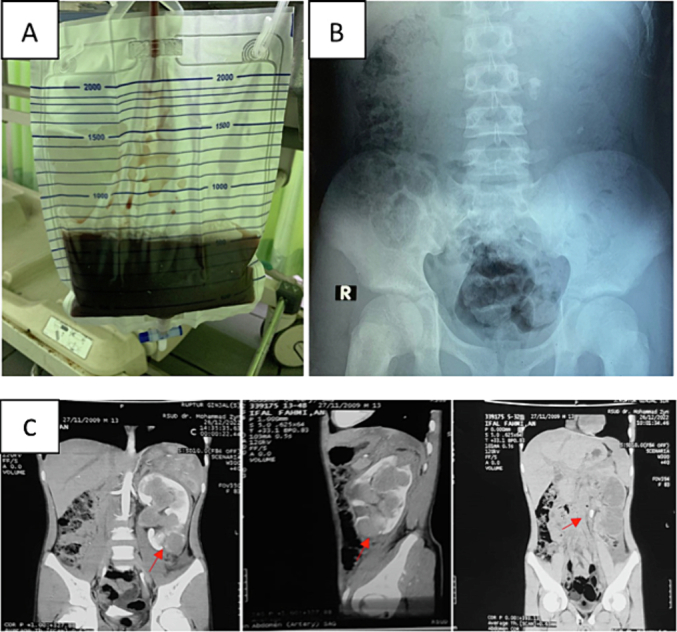
(A) Urine output was 250 cc/12 h with gross hematuria; (B) On abdominal X-ray examination was found multiple radiopaque shadows with the size of 1.4 × 0.7 cm at the VL 3–4 level on the left side; (C) Abdominal CT scan with intravenous pyelogram (IVP) revealed severe hydronephrosis with hydroureter and multiple stones in the left lower calyx pole measuring 0.6 cm (880 HU), as well as stones in the ureteropelvic junction (UPJ) (1182 HU).

The 13-year-old patient presented with left lower back pain and hematuria, which began after being elbowed in the left waist. The patient also reported nausea and vomiting. The patient had a history of weakness and pallor for three months before the incident. Hemodynamics were stable. Urine output was 250 cc/12 h or 41.67 mL/kg/h with gross hematuria (Fig. 2A). The complete blood count and renal function showed normal results. On abdominal X-ray examination, multiple radiopaque shadows were found, with a size of 1.4 × 0.7 cm, at the VL 3–4 level on the left side (Fig. 2B). Ultrasound examination showed moderate hydronephrosis. Abdominal CT scan with intravenous pyelogram (IVP) revealed severe hydronephrosis with hydroureter and multiple stones in the left lower calyx pole measuring 0.6 cm (880 HU), as well as stones in the ureteropelvic junction (UPJ) (1182 HU) (Fig. 2C). The patient underwent *retrograde pyelography-ureteroscopy* (RPG-URS) on the left side and insertion of a double-J (DJ) stent on the left side, followed by bed rest for 14 days. After several routine follow-up visits, the patient did not present with significant complaints.

### 2.3 Third patient

**Fig. 3:**
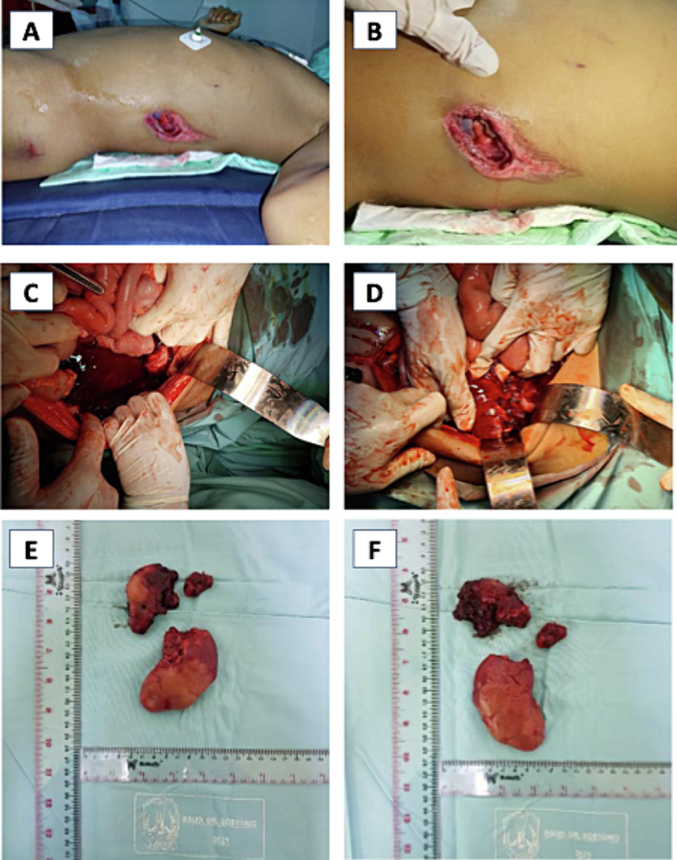
(A&B) Clinical picture wound on the left flank at the posterior axillary line; FAST results showed free fluid in Morrison's pouch and paracolic gutter; (C, D, E, F) Clinical picture rupture of the top pole of the left pyelum kidney and bleeding from a pedicle that was about 1.5 cm from the aorta.

After being involved in a motorcycle accident, a male 14-year-old came to our emergency room. He was riding with a helmet when he fell to the left side due to a slippery street. He was stabbed in the flank by a cutting tree trunk. On examination, the patient had a heart rate of 151 beats per minute, muscular defense, and reddish urine production of 400 mL over 15 h or 48.48 mL/kg/h. His Glasgow Coma Scale score was recorded as follows: eye response 3, verbal response 4, and motor response 5, with a total of 15. He had a puncture wound on his left flank at the posterior axillary line measuring 4 × 3 cm with apparent kidney exposure and an abrasion on his left trochanter (Fig. 3A &B). Resuscitation involving airway, breathing, and circulation management was promptly performed on this patient. Intravenous crystalloid fluids were immediately administered through double IV access. FAST results showed free fluid in Morrison's pouch and paracolic gutter. Laboratory results showed hemoglobin of 6.9 and leukocytes of 17.5. Following a surgical resuscitation and emergency exploratory laparotomy, the patient had internal bleeding from a rupture of the top pole of the left pyelum kidney and bleeding from a pedicle that was about 1.5 cm from the aorta (Fig. 3C, D, E, F). The patient underwent a left nephrectomy and received a packed red cell transfusion, antibiotics, and postoperative close monitoring. Patient discharge on 3rd day post-operation, creatinine evaluation one month post-surgery was normal.

### 2.4 Fourth patient

**Fig. 4:**
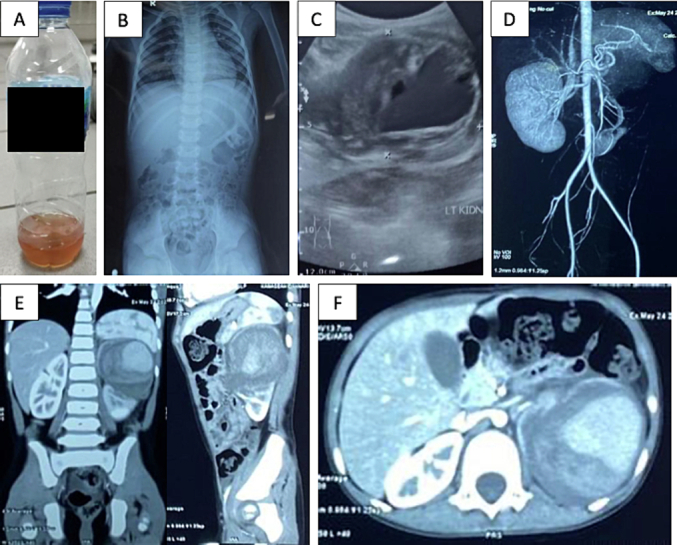
(A) Physical examination showed no hematuria; (B), Babygram results were expected; (C) ultrasound imaging revealed an aneurysm in the left kidney; (D, E, F) Abdominal CT angiography confirmed the presence of a hematoma at the upper pole with a pseudoaneurysm connected to a branch of the left renal artery.

A 4-year-old boy was referred to the emergency department from a secondary hospital due to recurrent hematuria and a history of anuria following blunt renal trauma. The patient had experienced six episodes of hematuria, anuria, and left flank pain after hitting the corner of a table while playing four months prior. Physical examination of the abdominal region revealed dull pain in the flank area, and the patient experienced moderate to severe abdominal tenderness upon palpation. The patient had undergone blood clot evacuation twice for blood clot intra-bladder and recurrent hematuria; he was referred to our hospital for angioembolization to manage the pseudoaneurysm. Laboratory examination results were within normal limits. Physical examination showed no hematuria (Fig. 4A), and urinalysis showed erythrocyte (3+) and leukocyte (2+). Babygram results were normal (Fig. 4B), while ultrasound imaging revealed an aneurysm in the left kidney (Fig. 4C). Abdominal CT angiography confirmed the presence of a hematoma at the upper pole with a pseudoaneurysm connected to a branch of the left renal artery (Fig. 4D, E, F). The patient underwent angioembolization and was observed for clot retention. The surgery was uneventful, and the patient was discharged without complications. Follow-up at six months postoperatively showed no functional or anatomical defects.

Management approaches varied based on the severity and nature of the genitourinary tract trauma in each case, including conservative management, surgical intervention, and minimally invasive procedures like angioembolization. Close monitoring and follow-up were emphasized to ensure proper recovery and address potential complications.

## 3 Discussion

Trauma is the leading cause of morbidity and mortality in children, with approximately 3 % of children assessed in pediatric hospital trauma departments having had trauma [12]. Trauma can result from blunt traumas such as falls, car crashes, sports injuries, physical assaults, or sexual abuse or from penetrating injuries such as gunshot or knife wounds. Of all blunt abdominal injuries resulting from anatomical causes, the kidney is the organ most frequently affected, accounting for about 10 % of cases [13]. Children are more prone than adults to experience kidney impairment following physical trauma because of their anatomy. Children are more susceptible than adults to renal injuries from blunt trauma because their kidneys are larger than those of adults and may still have fetal lobulations, which raises the likelihood of local parenchymal disruption. Children have weaker abdominal muscles and less perirenal fat, and their thoracic cage is less ossified, more elastic, and compressible than an adult's [14].

Studies show that 43 % of pediatric patient's blunt kidney trauma is caused by motor vehicle accidents, which are followed by falls from a height, pedestrian accidents, and sports injuries [1]. The child's body abruptly decelerating or coming into touch with blunt objects often causes these injuries. Deceleration or compressive traumas might cause the less well-protected adolescent renal parenchyma to contusion, lacerate, or avulse. The less well-protected pediatric renal parenchyma may contusion, lacerate, or avulse due to deceleration or compressive injuries. In our case series, penetrating trauma resulted in the third case, while blunt trauma from a car accident and a tree fall produced the first and second cases, respectively. The male-to-female ratio of 3:1 indicates that males are more likely than females to experience renal trauma, which is consistent with our case series in which all patients were male 2.

Several parameters, including the clinical examination, hemodynamic condition, extent of injury, concomitant organ injuries, and presence of hematuria, are considered when managing pediatric renal trauma [10]. However, in clinical practice, hemodynamic status and severity of renal trauma, according to AAST, were the primary considerations. A child with renal trauma is considered hemodynamically stable if their systolic blood pressure is above 90 mmHg plus twice their age of 11. In our case series, the first patient presented with grade 5 blunt pediatric renal trauma and stable hemodynamics. He was treated conservatively with close monitoring for 14 days. The second patient presented with grade 4 left renal trauma due to blunt trauma. He underwent *retrograde pyelography-ureteroscopy* (RPG-URS) on the left side and the insertion of a DJ stent on the left side for renal stone. Bed rest for 14 days was the conservative course of treatment for the traumatic kidney damage.

The standard approach to treating blunt kidney damage involves non-surgical conservative therapy, bed rest, fluids, and monitoring vital signs and hemoglobin/hematocrit levels [7]. It has been shown that this method works well even for children who have sustained high-grade renal damage. However, periodic reevaluations of the patient's general status, serial imaging, and careful clinical supervision are needed. Because hemodynamic instability might arise during non-operative therapy, hemodynamic stability is the critical management criterion for all renal injuries [8].

It is advised to save emergency surgical intervention for situations with hemodynamic instability. Minimally invasive techniques like stenting, angioembolization, and percutaneous drainage may be utilized when necessary [13]. Surgery is required when there is continuous bleeding into an unconfined or growing hematoma. Extensive urine extravasation and a large amount of non-viable renal tissue are two other related reasons for surgery [15]. Endourological procedures may often effectively control persistent extravasation or urinoma [16].

An example of surgical intervention in treating pediatric renal trauma is shown in the third instance. Patients experiencing hemodynamic instability should consider urgent surgical intervention. Relative indications include significant non-viable renal tissue and massive urine extravasation. Surgery is indicated in cases of chronic bleeding into a growing or unconfined hematoma [15]. In our third case, the patient had internal bleeding due to grade 5 renal trauma, and a nephrectomy was performed to remove the non-functioning kidney. Surgical intervention for unstable hemodynamic renal trauma is recommended to hasten recovery and reduce long-term complications in pediatric patients [17]. In addition to surgery, the patient received a blood transfusion to treat anemia caused by internal bleeding. Severe penetrating trauma typically requires a greater need for blood transfusion and a higher likelihood of nephrectomy.

The fourth case in point emphasizes the value of close observation when managing pediatric renal trauma non-operatively. Angioembolization, a surgical intervention, may be indicated for recurrent hematuria caused by a pseudoaneurysm. According to the literature, hemodynamic instability, persistent bleeding, and unconfined hematoma indicate emergent operative intervention [6]. The patient in this particular case had a grade 4 blunt renal trauma diagnosis and required angioembolization due to recurrent hematuria, unconfined hematoma, and a pseudoaneurysm. Pseudoaneurysms are rare and may present as secondary hematuria following renal trauma. This risk is highest in patients with higher-grade trauma who are managed conservatively [18,19]. Further evaluation may include renal angiography with selective angioinfarction of the bleeding site to minimize radiation exposure. Upon diagnosis, the standard treatment for renal arterial pseudoaneurysms is selective angioembolization [20].

Our case series depicts blunt and penetrating renal trauma in pediatric populations. Regarding the outcomes, penetrating injuries to the kidney are generally worse than blunt injuries because penetrating injuries tend to be more severe and involve other organs [21]. Acute complications are bleeding and infection, while rare complications are the development of an arteriovenous malformation or pseudoaneurysm, which manifests as secondary hematuria. The most significant late complications of renal trauma are hypertension and loss of renal function [6]. According to studies, grade 4 and 5 injuries are more likely to result in some permanent reduction in renal size and function [22,23]. However, the kidneys may sometimes retain their blood supply and viability and become realigned in a functional configuration. Long-term kidney function is mainly affected by persisting morphological changes, including scars, cysts, or segmental hydronephrosis.

## 4 Conclusion

The case series highlights the significance of treating pediatric renal trauma patients individually according to their hemodynamic status and degree of injury in line with current guidelines. When it comes to high-grade renal trauma, stable patients may benefit from conservative treatment and attentive observation, while those who are hemodynamically unstable may need surgical intervention. In cases of non-operative management, surgical intervention may be required if specific indications are present, such as hemodynamic instability and a pseudoaneurysm.

## Ethical approval

Ethical approval to report this case was obtained from The Hospital Research Ethics Committee of “Dr. Soetomo General Academic Hospital” where the patient was admitted.

## Funding

The authors received no financial support for the research, authorship and/or publication of this article.

## Author contribution

Soetojo Wirjopranoto: Conceptualization, Supervision, Methodology, Writing - original draft, Writing - review & editing, Validation.

Yufi Aulia Azmi: Conceptualization, Resources, Methodology, Project Administration, Writing - original draft, Investigation

Kevin Muliawan Soetanto: Conceptialization, Methodology, Writing – original draft.

## Guarantor

Soetojo Wirjopranoto

## Research registration number

1. Name of the registry: Not required
2. Unique identifying number or registration ID: Not required
3. Hyperlink to your specific registration (must be publicly accessible and will be checked): Not required

## Conflict of interest statement

The authors report no declarations of interest.

## Data Availability

No data was used for the research described in the article.
